# Children and adolescent patients with goiter and normal thyroid function: US findings related to underlying autoimmune thyroid diseases

**DOI:** 10.1097/MD.0000000000030095

**Published:** 2022-09-02

**Authors:** Sook Min Hwang, Ji-Young Hwang, Jin Hee Moon, Ik Yang, Ji Young Woo, Hye Jin Lee

**Affiliations:** a Department of Radiology, Kangnam Sacred Heart Hospital, Hallym University College of Medicine, Seoul, Korea; b Department of Pediatrics, Kangnam Sacred Heart Hospital, Hallym University College of Medicine, Seoul, Korea.

**Keywords:** adolescents, autoimmune thyroid diseases, children, echogenicity, thyroid, ultrasonography

## Abstract

This study was conducted to investigate and compare thyroid ultrasonography (US) findings in children and adolescents with goiter and normal thyroid function with positive or negative thyroid autoimmunity. From 2000 to 2020, we reviewed initial thyroid US images in 33 autoimmune thyroid diseases (AITDs) patients and 52 nonAITD patients. Our review of the images focused on thyroid parenchymal hypoechogenicity and heterogeneous echopattern subdivided into 2 groups according to severity: hypoechogenicity 1 and 2 (HO1 and HO2) and heterogeneity 1 and 2 (HE1 and HE2). HO1 and HE1 were observed more frequently in the nonAITD group (86.5% and 42.3%, respectively), while HO2 and HE2 were observed more frequently in the AITDs group (36.4% and 81.8%, respectively). More patients in the AITDs group showed change of both US groups and thyroid function state within the follow-up periods than in nonAITD group (33.3% and 5.77%, respectively). Children and adolescent AITDs patients showed more severe parenchyma hypoechogenicity and heterogeneous echopattern compared with nonAITD patients with goiter and normal thyroid function.

## 1. Introduction

Goiter is the most common physical presentation of thyroid disease in children and adolescents.^[1]^ Goiter is enlargement of the thyroid gland resulting from various causes including inflammation owing to AITDs, space occupying lesion, or infection.^[2]^ Among them, AITDs are the most common cause of thyroid dysfunction in children and adolescents.^[3]^

It is improper to predict underlying diseases that cause thyroid dysfunction with only goiter presentation because it is nonspecific and can be accompanied by normal or abnormal thyroid functions. Therefore, when goiter appears, the appropriate examination to be carried out depends on patient circumstance.

To identify underlying causes of goiter, several evaluations should be performed. When patients show hypo- or hyperthyroidism, thyroid autoantibody level should be assessed with suspicion of AITDs.^[3]^ In cases of patient with goiter and normal thyroid function, which is called euthyroidism, there is no consensus regarding choice of autoantibody evaluation.^[4]^

US is a widely used diagnostic tool for evaluating thyroid size, anatomy, and parenchymal abnormalities adjunct to the clinical exam.^[5]^ On US of patients with goiter, thyroid gland enlargement, parenchymal abnormality, or focal lesion can be found.^[5,6]^ Previous studies have analyzed thyroid parenchymal echogenicity on US among the general population and in patients with underlying Hashimoto disease (HT).^[7,8]^ Unfortunately, there is scarce previous study of US findings in children and adolescent patients with goiter and initial normal thyroid function. However, this situation should not be overlooked because thyroid dysfunction can appear later.^[9,10]^

The purpose of this study was to investigate and compare thyroid US findings of children and adolescents with goiter and normal thyroid function in patients with positive and negative thyroid autoimmunity.

## 2. Materials and Methods

### 2.1. Study population

This retrospective study was approved by our institutional review board. The requirement for informed consent was waived, and patient information was anonymized prior to analysis.

We completed a retrospective search of medical records using the electronic database from January 2000 through April 2020, which yielded 85 consecutive children and adolescent patients with goiter and normal thyroid function. All patients underwent initial thyroid US and biochemical evaluation of thyroid autoimmunity within 3 months after US examination. The AITDs group was comprised of 33 patients (male to female ratio, 3:30; mean age 15 ± 3.80 years) who were positive for thyroid autoimmunity including antithyroglobulin antibodies (TGAb), antithyroid peroxidase antibodies (TPOAb), or thyroid stimulating hormone (TSH) receptor antibodies (TRAb). The nonAITD group consisted of 52 patients (male to female ratio, 13:39; mean age 12 ± 3.9 years) who were negative for all 3 thyroid autoantibodies and regarded as the control group.

The patients’ medical charts were reviewed for demographics, clinical profiles, and detailed biochemical thyroid function tests.

### 2.2. US examinations

All patients underwent a detailed thyroid US examination, which was performed by 1 of 3 pediatric radiologists (three radiologists with 10, 17, and 18 years of experience in thyroid US, respectively, at the time of first assessment of study participants). The US systems (IU22 US or HDI 5000, Philips Healthcare; LogiQ E9, GE Healthcare) with high-resolution linear-array transducers (5–12 MHz and 6–15 MHz) were used for the examinations.

Two radiologists (with 10 and 12 years of pediatric imaging interpretation experience, respectively) retrospectively reviewed all images in consensus. Although the reviewers knew that all patients had been referred with a chief complaint of goiter, they were unaware of other clinical information.

**Figure 1.**
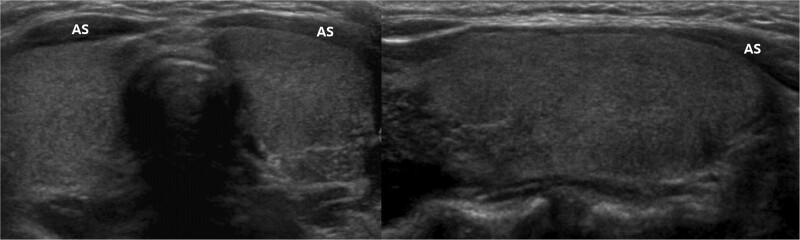
Transverse and longitudinal US images of the HO1 group, defined by lower parenchymal echogenicity than that of normal thyroid tissue but more echogenic compared to the anterior strap muscle (AS).

**Figure 2.**
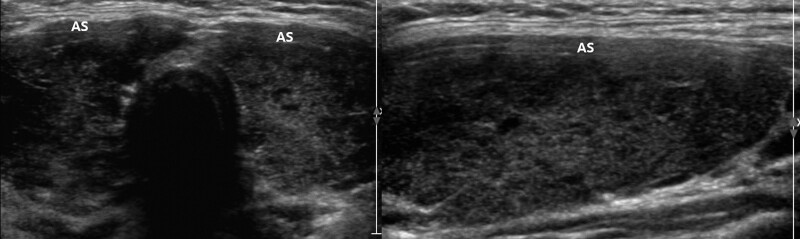
Transverse and longitudinal US images of the HO2 group, defined as more hypoechoic than the AS.

**Figure 3.**
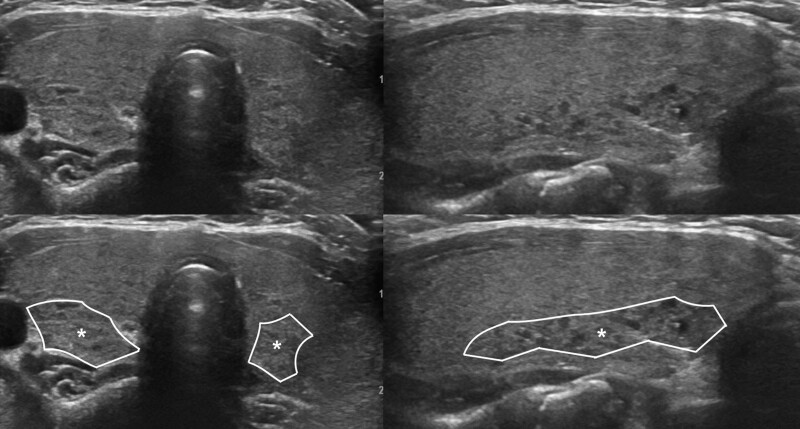
Transverse and longitudinal US images of HE1, defined as echogenicity area of irregular or mottled shape with or without internal echogenic strands involving <50% of the entire thyroid gland. Note the outlined area with internal asterisk in lower column images.

**Figure 4.**
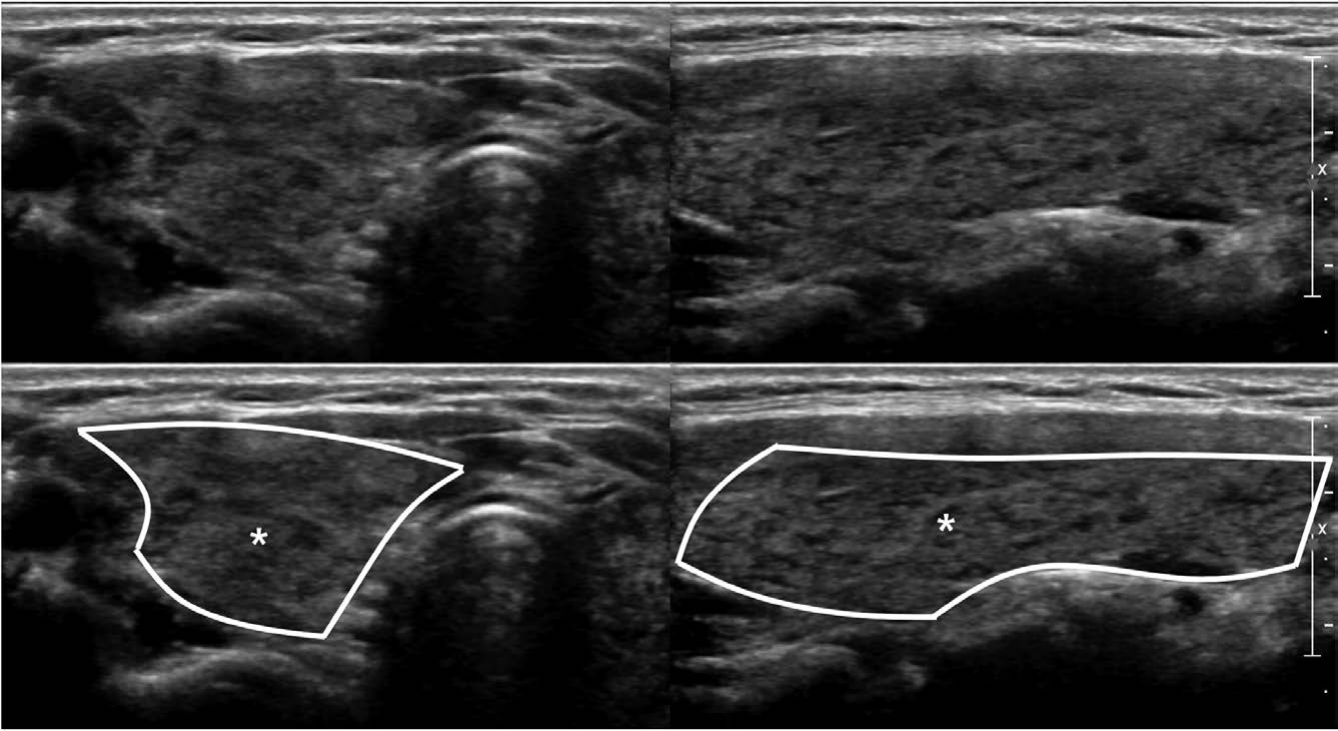
Transverse and longitudinal US images of the HE2 group, defined as echogenicity area of irregular or mottled shape with or without internal echogenic strands involving more than 50% of the entire thyroid gland. Note the outlined area with internal asterisk in lower column images.

The parameters assessed by US included thyroid gland enlargement, parenchymal hypoechogenicity, and parenchymal heterogeneous echopattern. Thyroid gland enlargement was defined when it exceeded the normal range according to height and weight of the patient.^[11]^ Parenchymal hypoechogenicity was according to degree of low echogenicity area within thyroid gland compared to the structure of the adjacent anterior strap muscle (AS). This type of hypoechogenicity was subdivided into 2 groups. Hypoechogenicity grade 1 (HO1) (Fig. 1) was defined by lower parenchymal echogenicity than that of normal thyroid tissue but more echogenic compared to the AS. Hypoechogenicity grade 2 (HO2) (Fig. 2) was defined by greater hypoechogenicity than the AS. Parenchymal heterogeneous echopattern also was subdivided into 2 groups. Heterogeneous grade 1 (HE1) (Fig. 3) was defined as echogenicity area of irregular or mottled shape with or without internal echogenic strands involving <50% of the entire thyroid glands. Heterogeneous grade 2 (HE2) (Fig. 4) was defined as echogenicity area of irregular or mottled shape with or without internal echogenic strands involving more than 50% of the entire thyroid glands. US grades were evaluated qualitatively by visual assessment.

### 2.3. Statistical analysis

Statistical analyses were performed using SPSS software (SPSS, version 23.0; SPSS, Chicago, IL, USA). Data were considered significant if the *P*-value was *<* .05. Descriptive data were summarized as the mean ± standard deviation or number of individuals (percentage). Statistical differences were compared using either 2-sample t-test or Mann-Whitney test for continuous variables and Fisher exact test for categorical variables.

## 3. Results

### 3.1. Clinical profiles and laboratory data

The clinical profiles and laboratory data in AITDs and nonAITD groups are presented in Table 1. Both groups reflected female predominance. TGAb and TPOAb levels in the AITDs group were significantly higher than those in the nonAITD group.

**Table 1:** The clinical profiles and laboratory data in AITDs and Non AITD groups.

Variable	Total (N = 85)	AITDs (n = 33, 38.8%)	Non AITD (n = 52, 61.2%)	*P*-value
Age (year)	13 ± 4.1	15 ± 3.8	12 ± 3.9	0.873
Male	16 (18.8 %)	3 (9.1 %)	13 (25 %)	1.000
Female	69 (81.2 %)	30 (90.9 %)	39 (75 %)	
TSH (0.55–0.478 uIU/mL)	2.47 ± 2.18	2.33 ± 3.00	2.55 ± 1.46	0.028
T3 (60–181 ng/dL)	116.61 ± 30.95	108.01 ± 33.53	122.17 ± 28.13	0.045
fT4 (0.89–1.76 ng/dL)	1.24 ± 0.22	1.22 ± 0.18	1.26 ± 0.24	0.264
TGAb (5–100IU/mL)	216.42 ± 486.76	407.03 ± 629.28	17.86 ± 25.19	<0.001
TPOAb (1–16IU/mL)	710.52 ± 1265.07	1265.67 ± 1475.1	1.16 ± 2.25	<0.001
TRAb (0.0–1.75IU/L)	6.59 ± 15.73	6.59 ± 15.73	NA	

Values are presented as mean ± standard deviation (range) or number (%).

AITDs = autoimmune thyroid diseases, fT4 = Free throxine, TGAb = antithyroglobulin antibodies, TPOAb = antithyroid peroxidase antibodies, TRAb TSH receptor antibodies, TSH = thyroid stimulating hormone, T3 = triiodothyronine.

### 3.2. Imaging findings

**Figure 5.**
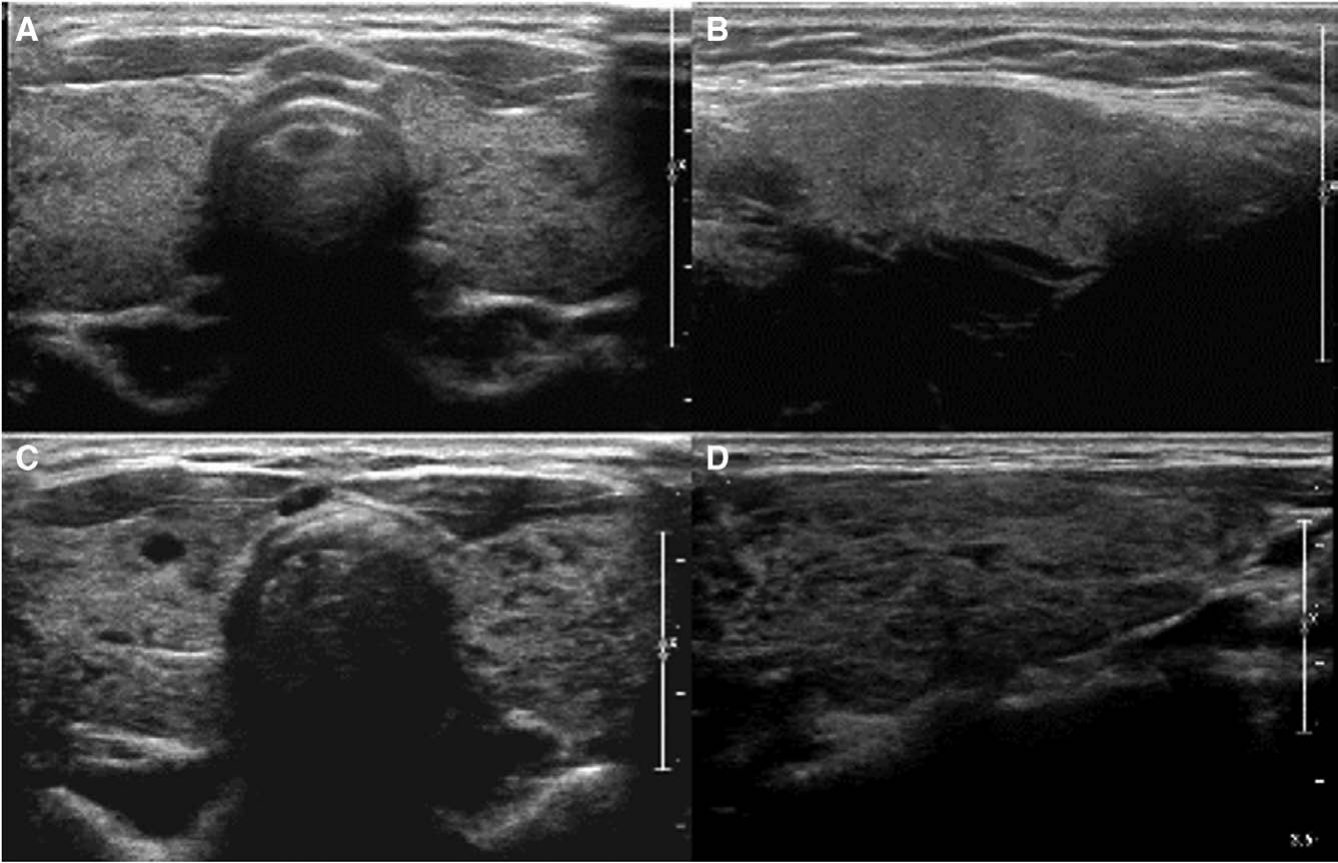
Transverse and longitudinal US images of the patient number 3 on Table 3. On initial US (a, b) thyroid echogenicity was classified into HO1/HE1 group. On follow up US after 20 months (c, d) thyroid echogenicity was aggravated and classified into HO2/HE2 group. At the same time thyroid function was aggravated as well from initial euthyroidism to subclinical hyperthyroidism.

The US findings of the 2 groups are presented in Table 2. All patients in both groups showed enlarged thyroid gland on US. HO1 was observed more frequently in the nonAITD group (45/52 86.5%) than in the AITDs group (21/33 63.6%) (*P* = .017). HO2 was observed more frequently in the AITDs group (12/33 36.4%) than in the nonAITD group (7/52 13.5%) (*P* = .031). HE1 was observed more frequently in the nonAITD group (22/52 42.3%) than in the AITDs group (6/33 18.2%) (*P* = .0322). HE2 was observed more frequently in the AITDs group (27/33 81.8%) than in the nonAITD group (30/52 57.7%) (*P* = .032). There were 11 out of 33 patients (33.3%) who showed change of US group in the AITDs group and 3 out of 52 patients (5.77%) in the nonAITD group (Table 3). Thyroid hormone level was also changed in patients who showed change in US group, from normal to subclinical hypo/hyperthyroidism or overt hypo/hyperthyroidism (Fig. 5). There were significantly more patients with change in both US groups and thyroid function states in the AITDs group (*P* = .001). The mean follow-up period in the AITDs group was 22.8 ± 2.18 months, and that of the nonAITD group was 15.7 ± 13.9 months.

**Table 2:** The US findings in AITDs and Non AITDs groups.

	AITDs (n = 33)	NonAITD (n = 52)	*P*
Enlarged	33 (100%)	52 (100%)	1.000
HO1	21 (63.6%)	45 (86.5%)	0.0175
HO2	12 (36.4%)	7 (13.5%)	0.0315
HE1	6 (18.2%)	22 (42.3%)	0.0322
HE2	27 (81.8%)	30 (57.7%)	0.0322

Values are presented as number (%).

AITDs = autoimmune thyroid diseases, HO = hypoechogenicity, HE = heterogeneous echopattern.

**Table 3:** The US findings and thyroid status change at follow up in AITDs and NonAITD groups.

	Patient number	US grades	Thyroid function states	p
AITD (n = 11, 33.3%)	1	HO1/HE2 → HO2/HE2	Eu → overt hyperthyroidism	0.0016
	2	HO1/HE2 → HO2/HE2	Eu → subclinical hyperthyroidism	
	3	HO1/HE1 → HO2/HE2	Eu → subclinical hyperthyroidism	
	4	HO1/HE2 → HO2/HE2	Eu → overt hyperthyroidism	
	5	HO1/HE2 → HO2/HE2	Eu → overt hyperthyroidism	
	6	HO1/HE2 → HO2/HE2	Eu → overt hypothyroidism	
	7	HO1/HE2 → HO2/HE2	Eu → overt hyperthyroidism	
	8	HO1/HE2 → HO2/HE2	Eu → overt hypothyroidism	
	9	HO1/HE1 → HO2/HE2	Eu → subclinical hypothyroidism	
	10	HO1/HE1 → HO2/HE2	Eu → subclinical hypothyroidism	
	11	HO1/HE2 → HO2/HE2	Eu → subclinical hypothyroidism	
Non (n = 3. 5.77%)	1	HO1/HE1 → HO2/HE2	Eu → subclinical hyperthyroidism	
	2	HO1/HE1 → HO2/HE2	Eu → subclinical hypothyroidism	
	3	HO1/HE1 → HO2/HE2	Eu → subclinical hypothyroidism	

AITDs = autoimmune thyroid diseases, EU = euthyroidism, HE = heterogeneous echopattern, HO = hypoechogenicity.

## 4. Discussion

Our results revealed that children and adolescent patients with goiter and euthyroidism in the AITDs group showed more severe hypoechogenicity and heterogeneous echopattern of thyroid gland on initial US than did those in the non AITD group. In addition, in the AITDs group, thyroid dysfunction was more frequently observed, and the US findings were aggravated during follow-up.

In children and adolescents, goiters are often found incidentally by parents or doctors.^[1]^ When goiter presents, thyroid abnormality can be considered first because it is the most common endocrinologic problem in children and adolescents.^[12]^ In children and adolescent patients with goiter, presence or absence of symptoms and degree of thyroid dysfunction are variable. Even in goiter patients with risk of underlying thyroid disease with positive thyroid autoantibody, in a euthyroid state, symptoms might not be apparent. Onset of thyroid function abnormality and symptoms are challenging to predict. As a result, in children and adolescent thyroid dysfunction patients, diagnosis and treatment can be delayed.^[1]^ Normal thyroid function is especially important in children and adolescents not only for physical development, but also for intellectual and cognitive development.^[13]^

The most common underlying cause of goiter is AITDs, including HT and Graves’ disease (GD), which are the most common causes of hypothyroidism and hyperthyroidism in children and adolescents, respectively.^[6,7,15,16]^ HT is a goitrous form of AITD. In HT, a high level of antithyroid antibodies damage the thyroid gland cells with infiltration by macrophages and lymphocytes.^15–17]^ In GD, autoantibodies against the TSH receptor on follicular epithelial cells mimic TSH and stimulate the thyroid gland. This results in overproduction of thyroid hormone.^[18]^ Histologically, lymphoplasmacytic infiltration, germinal center formation, and follicular destruction are seen in HT; and hypercellular, patchy lymphocytes infiltration and a decrease in colloid amount are noted in GD.^[19]^

For diagnosis of AITDs, various tests can be performed, among which the presence of thyroid autoimmunitiy is confirmatory. US is often used to identify thyroid parenchymal pattern change, size change, and presence of focal lesions.^[6,15,20]^ In US of AITD patients, thyroid glands tend to be enlarged with hypoechogenicity, and uneven parenchymal echogenicity can present as well.^[6]^ Pathologically, decrease in colloid content, lymphocyte infiltration among thyroid follicles, variable degree of fibrosis, and increase in intrathyroidal flow change the normal follicular structure of the thyroid parenchyma in AITD patients.^[21,22]^ Because thyroid gland tissue echogenicity varies according to cellularity and vascularization,^[23–25]^ the US pattern of the thyroid gland in AITD patients can change as mentioned above.

There were several studies with AITD patients or general population evaluating usefulness of thyroid US for detection of disease. Rago T. et al, found that hypoechogenicity on thyroid US can detect thyroid autoimmune disease in apparently healthy patients in advanced cases, while present and future thyroid dysfunction are more rapidly predicted by hypoechogenicity via US than by presence of serum thyroid autoantibodies in adults.^[26]^ Jeong SH et al, reported that hypoechogenicity grade of the thyroid gland correlates with HT severity in children and adolescents.^[7]^ According to Pernille V et al, among healthy populations not diagnosed with existing thyroid abnormalities, there is tendency for future thyroid dysfunction when heterogeneity is present on initial US.^[8]^

In cases of children and adolescent euthyroid goiter, about 20% develop AITDs presenting of hyper- or hypothyroidism requiring future treatment. There are studies on factors that can predict thyroid dysfunction in patients with euthyroid goiter which should not be overlooked because thyroid function abnormality appears in a considerable proportion of such patients. In adult goiter patients, treatment is recommended when antibodies are identified.^[6]^ In children and adolescents who have euthyroid goiter, it is helpful to assess antibody levels to predict the need for future treatment. It was also reported that, in initial antibody-negative euthyroid goiter patients, progression to HT during follow up was seen frequently in patients with family history or persistent goiter over an average follow up period of about 2 years.^[4]^ In our study, the mean follow-up periods between initial thyroid US and biochemical thyroid dysfunction with changes in US groups were about 22 and 15 months in AITDs and nonAITD groups, respectively. Considering previous study and our results, it is better to advise thyroid function test and US follow up for at least 2 years to monitor development of thyroid dysfunction in euthyroid goiter children and adolescent patients regardless of autoantibody presence.

Autoimmue thyroid disease was reported more prevalent during developing of secondary sex characteristics and at puberty.^[27]^ The age of development of secondary sex characteristics ranges 9–13 years in girls and 10–14 years in boys.^[28]^ The average age of menarche onset in Koreans was 12.7 year.^[29]^ The mean age in our study was 15 years in AITDs group and 12 years in nonAITD group, which implies most of the children in our study were at the age of secondary sexual characteristics development. For AITDs group, the average age was about 3 years higher than nonAITD group, so more children with developed secondary sexual characteristics, such as the onset of menarche, would have been included. In addition, the incidence of AITDs and thyroid dysfunction increases with age.^[30]^ The physician should investigate symptoms of hyper/hypothyroidism more carefully and frequently in post-pubertal adolescents than in prepubertal children.

In our study, children and adolescent goiter patients with initial normal thyroid function and negative autoantibody test also showed increase in thyroid gland size, mild hypoechogenicity, and heterogeneous echopatterns on US. The pathogenesis of thyroid gland enlargement is not clear in this case. Histologically, increased thyroid follicle size filled with a large amount of colloid was noted. There was no combined inflammation, infection, or neoplasm in enlarged thyroid gland.^[12]^ In congenital hypothyroidism, possible genes for euthyroid goiters have been noted.^[31]^ However, in our study, the target patients are children and adolescents, so it is difficult to see them as hereditary. At puberty, the thyroid gland tends to be enlarged in patients with antibody-negative goiter.^[32]^ In girls, timing of puberty is similar to that of increase in thyroid size. In our study, thyroid gland enlargement and mild change in parenchyma in the nonAITD patients group was explained partially as above.

In our study, initial TPOAb and TRAb levels were significantly higher in the AITDs group. Various conclusions have been drawn in previous studies on whether thyroid antibody level is related to degree of US findings. In a few studies, there was no correlation between autoantibody level and thyroid gland echogenicity.^[33]^ However, in cases of mild thyroid dysfunction, together with US, thyroid antibody levels are helpful to predict the future direction of the disease and the need for treatment.^[34]^ Since our study was conducted in patients with normal thyroid function, we suggest that we are now closer to confirming that measuring initial antibody levels are helpful in mild thyroid dysfunction cases.

In our study, TGAb was positive in 53.8% (14/26) in AITD group and 3.8% (1/26) in nonAITD group, respectively. TPO was positive in 75% (18/24) in AITDs group and 30.8% (8/26) in nonAITD group, respectively. According to previous study in general population of adults, TGAb was positive in 27.9 ~72.7% and TPO was positive in 32.1~72.7%, respectively.^[8]^ In AITDs group, TGAb showed similar result and TPOAb showed more prevalent result. In nonAITD group, TGAb showed less prevalent result and TPOAb showed similar result. In previous study with adults, as the thyroid gland echogenicity decreased, the positive antibody rate increased as well.^[8]^ In our study, the higher positivity rate of TPOAb in the AITDs group may be due to the fact that many patients with a higher frequency and severity of hypoechogenicity were included.

Our study has several limitations. First, our study was retrospective study with only 20 years limited data and had small sample size in both groups. Although all US examinations were performed by qualified pediatric radiologists according to a detailed US protocol, prospective study with a larger-scale investigation is necessary to validate US findings. Second, there may had been a bias because radiologists knew that patient had thyroid ultrasound with suspicion of goiter. Third, there was limited follow-up duration, which might have missed later development of thyroid dysfunction.

## 5. Conclusions

Our study showed that US findings can predict the association with AITDs in children and adolescent patients with normal thyroid function who presented with the first physical presentation of goiter without previous thyroid abnormalities. This can help in the decision of further evaluation and management in patients with goiter and normal thyroid function.

## Author contributions

Conceptualization: Sook Min Hwang.

Data curation: Sook Min Hwang, Ji-Young Hwang, Jin Hee Moon, Ik Yang, Ji Young Woo.

Formal analysis: Sook Min Hwang.

Investigation: Sook Min Hwang, Hye Jin Lee.

Methodology: Sook Min Hwang.

Project administration: Sook Min Hwang, Hye Jin Lee.

Resources: Sook Min Hwang, Hye Jin Lee.

Validation: Sook Min Hwang.

Visualization Sook Min Hwang.

Writing - Original Draft: Sook Min Hwang.

Writing - Review and Editing: Sook Min Hwang, Hye Jin Lee.

Sook Min Hwang orcid: 0000-0003-1755-4846.

## References

[1] Patrick H Katherine L Andrew JB. Thyroid disorders in children and adolescents A review. JAMA Pediatr. 2016;170:1008–19. 27571216 10.1001/jamapediatrics.2016.0486

[2] World Health Organization. A Guide for Programme Managers. Assessment of Iodine Deficiency Disorders and Monitoring their Elimination. 3rd ed. Geneva, Switzerland 2007;24–46.

[3] Biatta S Amir AB. Thyroid ultrasound part 1: technique and diffuse disease. Radiol Clin North Am. 2011;49:391–416. 21569900 10.1016/j.rcl.2011.02.002

[4] Kim SY Lee YA Jung HW. Pediatric Goiter: can thyroid disorders be predicted at diagnosis and in follow-up? J Pediatr. 2016;170:253–9.e1. 26706234 10.1016/j.jpeds.2015.11.008

[5] Laszlo H. Thyroid ultrasonography as a screening tool for thyroid disease. Thyroid. 2004;14:879–80. 15671764 10.1089/thy.2004.14.879

[6] Mamatha K Anshu G Uma RP. Ultrasound characteristics of the thyroid in children and adolescents with Goiter: a single center experience. Thyroid. 2015;25:176–82. 25340407 10.1089/thy.2014.0161 PMC4322035

[7] Jeong SH Hong HS Lee JY. The association between thyroid echogenicity and thyroid function in pediatric and adolescent Hashimoto’s thyroiditis. Medicine Baltimore. 2019;98:e15055. 30946351 10.1097/MD.0000000000015055 PMC6455779

[8] Pernille V Nils K Hans P. The association between hypoechogenicity or heterogeneity at thyroid ultrasonography and thyroid function in the general population. Eur J Endocrinol. 2006;155:547–52. 16990653 10.1530/eje.1.02255

[9] Sato A Aizawa T Koizumi Y. Ten-year follow-up study of thyroid function in euthyroid patients with simple goiter or Hashimoto’s thyroiditis. Intern Med. 1995;34:371–5. 7647404 10.2169/internalmedicine.34.371

[10] Effraimidis G Wiersinga WM. Mechanisms in endocrinology: autoimmune thyroid disease: old and new players. Eur J Endocrinol. 2014;170:R241–52. 24609834 10.1530/EJE-14-0047

[11] Marilyn JS. Pediatric Sonography. 4th ed. Philadelphia: Lippincott Williams & Wilkins, 2010.

[12] Sarah M. Diagnostic Approach to Goitre in Children. Vol 6. Ottawa: Ontario, 2001. 10.1093/pch/6.4.195 PMC2804541 20084235

[13] Susan RR Rosalind SB Lawson W. Update of newborn screening and therapy for congenital hypothyroidism. Pediatrics. 2006;117:2290–303. 16740880 10.1542/peds.2006-0915

[14] Elpis V Dimitrios T Feneli K. Evolution of sonographic appearance of the thyroid gland in children with Hashimoto’s thyroiditis. J Pediatr Endocrinol Metab. 2009;22:339–44. 19554808 10.1515/jpem.2009.22.4.339

[15] Elizabeth NP Alan PF Lewis EB. Thyroiditis. N Engl J Med. 2003;348:2646–55. 12826640 10.1056/NEJMra021194

[16] Kabelitz M Liesenkötter KP Stach B. The prevalence of antithyroid peroxidase antibodies and autoimmune thyroiditis in children and adolescents in an iodine replete area. Eur J Endocrinol. 2003;148:301–7. 12611610 10.1530/eje.0.1480301

[17] Kucharska AM Witkowska-Sȩdek E Labochka D. Clinical and biochemical characteristics of severe hypothyroidism due to autoimmune thyroiditis in children. Front Endocrinol (Lausanne). 2020;11:364. 32733376 10.3389/fendo.2020.00364 PMC7360718

[18] Rivkees SA. Controversies in the management of graves′ disease in children. J Endocrinol Invest. 2016;39:1247–57. 27153850 10.1007/s40618-016-0477-x

[19] Lester DR. Diffuse hyperplasia of the thyroid gland (Graves’ disease). Ear Nose Throat J. 2007;86:666–7. 18225623

[20] Manfred B Kenneth RF Bradley A. Ultrasonography of the thyroid. In: Endotext [Internet]. South Dartmouth (MA): MDText.com, Inc., 2000–2020 Apr 11.

[21] Pinchera A Fenzi GF Bartalena L Chiovato L Marcocci C. Thyroiditis. M De Vissher, ed. In: The Thyroid Gland. New York: Raven Press, 1980;413–441.

[22] Fisher DA Beall N. Hashimoto’s thyroiditis. In: Hershmann JM Bray GA, ed. New York: Pergamon Press, 1979;487–500.

[23] Muller HW Schroder S Schneider C. Sonographic tissue characterization in thyroid gland diagnosis. Klinische Wochenschrift. 1985;63:706–10. 3900555 10.1007/BF01733114

[24] LiVolsi VA. Surgical Pathology of the Thyroid. Vol 22. Philadelphia 1990.

[25] Ralls PW Mayekawa DS Lee KP. Color-flow Doppler sonography in Graves’ disease: “thyroid inferno”. Am J Roentgenol. 1988;150:781–4. 3279732 10.2214/ajr.150.4.781

[26] Rago T Chiovato L Grasso L. Thyroid ultrasonography as a tool for detecting thyroid autoimmune diseases and predicting thyroid disfunction in apparently healthy subjects. J Endocrinol Invest. 2001;24:763–9. 11765045 10.1007/BF03343925

[27] Skarpa V Kousta E Tertipi A. Epidemiological characteristics of children with autoimmune thyroid disease. Hormones (Athens). 2011;10:207–14. 22001131 10.14310/horm.2002.1310

[28] Susman EJ Houts RM Steinberg L. Longitudinal development of secondary sexual characteristics in girls and boys between ages 91/2 and 151/2 Years. Arch Pediatr Adolesc Med. 2010;164:166–73. 20124146 10.1001/archpediatrics.2009.261 PMC2863107

[29] Lee MH Kim SH Oh MK. Age at menarche in Korean adolescents: trends and influencing factors. Reprod Health. 2016;13:121. 27662834 10.1186/s12978-016-0240-y PMC5035449

[30] Dayan CM Daniels GH. Chronic autoimmune thyroiditis. N Engl J Med. 1996;335:99–107. 8649497 10.1056/NEJM199607113350206

[31] Krohn K Fuhrer D Bayer Y. Molecular pathogenesis of euthyroid and toxic multinodular goiter. Endocr Rev. 2005;26:504–24. 15615818 10.1210/er.2004-0005

[32] Fleury Y Melle GV Woringer V. Sex-dependent variations and timing of thyroid growth during puberty. J Clin Endocrinol Metab. 2013;86:750–4. 10.1210/jcem.86.2.7209 11158041

[33] Uwe S Wolfgang A Jan WK. Relationship of clinical features and laboratory parameters to thyroid echogenicity measured by standardized grey scale ultrasonography in patients with Hashimoto’s thyroiditis. Med Sci Monit. 2003;9:Mt13–17. 12709678

[34] Shin DY Kim EK Lee EJ. Role of ultrasonography in outcome prediction in subclinical hypothyroid patients treated with levothyroxine. Endocr J. 2010;57:15–22. 19823000 10.1507/endocrj.k09e-154

